# Mechanisms and Performance of a Copper‐Modified Lead Dioxide Anode for Electrochemical Cephalexin Removal: Roles of Hydroxyl Radicals and Operational Parameters

**DOI:** 10.1002/open.70256

**Published:** 2026-07-15

**Authors:** Haijie Li, Yuguang Chang, Qing Wei, Jingjing Cao

**Affiliations:** ^1^ School of Environmental Science Nanjing XiaoZhuang University Nanjing China; ^2^ Jiangsu Radiation Environmental Protection Consultation Limited Company Nanjing China

**Keywords:** antibiotic pollution control, electrochemical advanced oxidation process, hydroxyl radical, wastewater treatment

## Abstract

Cephalexin (CEX), a widely used β‐lactam antibiotic, is frequently detected in pharmaceutical and municipal wastewater and requires efficient removal before discharge. In this study, a Cu‐modified PbO_2_ anode (Cu/PbO_2_) was fabricated for electrochemical advanced oxidation of CEX. Compared with pristine PbO_2_, Cu/PbO_2_ showed a 2.35‐fold higher voltammetric charge (q*) and a lower charge–transfer resistance (17.5 vs. 21.2 Ω cm^2^), indicating more accessible electroactive sites and faster interfacial electron transfer. Under optimized conditions, CEX removal reached 70.9 ± 1.2% at 20 mA cm^−2^ within 120 min; increasing the current density to 30 mA cm^−2^ decreased the apparent rate constant from 0.0110 to 0.0092 min^−2^. Acidic conditions favored CEX degradation, with removal increasing from 64.5 ± 0.5% at pH 9 to 80.2 ± 1.1% at pH 3. Chloride showed a concentration‐dependent dual effect: 0.5–1.0 mM Cl^−^ enhanced CEX oxidation through reactive chlorine species, whereas 2–3 mM Cl^−^ inhibited degradation by competing for surface sites and scavenging hydroxyl radicals. Radical‐quenching experiments confirmed that hydroxyl radicals (^•^OH) were the dominant oxidants. Overall, Cu modification improves the activity and durability of PbO_2_ anodes and offers a practical design strategy for electrochemical treatment of antibiotic‐contaminated wastewater.

## Graphical abstract

A simple Cu modification transforms the performance of PbO_2_ anodes. Increased electroactive surface area, accelerated charge transfer, and enhanced hydroxyl‐radical utilization drive efficient cephalexin degradation while improving electrode durability. The findings reveal how interfacial engineering governs electrochemical oxidation and antibiotic removal in complex water matrices.

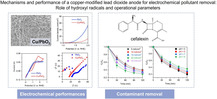

## 1 Introduction

Antibiotics have been widely used in human medicine, veterinary treatment, aquaculture, and livestock production to prevent and treat bacterial infections. It was reported that approximately 162,000 tons of antibiotics were consumed globally in 2013 [1], leading to their widespread presences. In this case, the antibiotics resistance gradually occurred in bacterial strains due to the upregulated gene expressions related to resistance when subinhibitory concentrations of antibiotics coexisted with bacterial [2]. Further, these antibiotics probably derives from effluent of pharmaceutical factories and hospitals [3], posing challenges to their efficient decontamination via traditional wastewater treatments and highlighting the need for the development of advanced technology to address the intractable problems. Cephalexin (CEX), a first‐generation cephalosporin, is widely prescribed for respiratory, urinary‐tract, skin, and soft‐tissue infections. Because antibiotics remain biologically active at low concentrations, their continuous discharge may impose selective pressure on microorganisms, disturb aquatic microbial communities, and accelerate the spread of antibiotic‐resistant bacteria and antibiotic‐resistance genes. Although no universal discharge limit for CEX has been established in most jurisdictions, its environmental persistence and biological activity highlight the need for efficient advanced treatment technologies [4, 5, 6].

Conventional biological treatment can partially remove some antibiotics, but β‐lactam antibiotics and their transformation products may persist because of their complex structures, inhibitory effects on microorganisms, and variable biodegradability. Adsorption is simple and energy saving, but it mainly transfers contaminants to a spent solid phase that requires regeneration or disposal. Membrane filtration provides high separation efficiency; however, fouling, concentrate management, and pressure‐related energy consumption restrict its standalone application. Photocatalysis, ozonation, Fenton‐like oxidation, and persulfate activation can degrade antibiotics through reactive oxygen species (ROS), but they often require external reagents, irradiation, catalyst recovery, or strict pH control (Table 1) [7, 8, 10, 11].

**TABLE 1:** Comparison of representative technologies for cephalexin removal and their cost‐related considerations.

Technology	Conditions	Removal	Cost/energy	Ref.
Adsorption on MCM‐41	CEX 50 mg L^−1^; pH 3; 800 mg L^−1^ adsorbent; 40 °C; 30 min	90.3% removal	/	[7]
Fe–TiO_2_/Bi_2_O_3_ photocatalysis	CPX 5 mg L^−1^; 1.5 g L^−1^ catalyst; pH 9; visible LED 240 min or UV 120 min	74% visible; 96% UV	/	[8]
Photocatalytic ozonation	N‐TiO2/ZnFe_2_O_4_/zeolite + O_3_ + visible light	95% CEX degradation	/	[9]
SDS‐modified Ti/TiO_2_/β‐PbO_2_	CEX 50 mg L^−1^; 20 mA cm^−2^; pH 7.01; 20 min	97.6% degradation; 70.27% COD removal	0.23 × 10^−3^ kWh mg^−1^	[10]
Cu/PbO_2_	CEX 1 mM; 100 mM Na_2_SO_4_; 20 mA cm^−2^; pH 7; 120 min	70.9 ± 1.2%; 80.2 ± 1.1% at pH 3; 68.2 ± 2.1% in actual wastewater	0.24 kWh m^−3^	This study

Recently, advanced oxidation processes (AOPs) have attracted growing interest as a promising alternative due to its abundant ^•^OH production, high efficiency, and excellent performances in contaminant removal. The powerful ^•^OH (2.80 V vs. SCE) as a nonselective and short‐life reactive specie (half life time: ∼1 ns) [12, 13] is able to react with the contaminants and mineralizes them into CO_2_, H_2_O, and inorganic ions [14]. Specifically, electrochemical advanced oxidation processes (EAOPs) as an emerging wastewater treatment technology with advantages such as no secondary contaminations, flexible operational scale, and tunable current parameters [13]. In EAOPs, there are two main pathways for pollutants degradation, namely, (i) direct oxidation through electrons transfer on anodic surface and (ii) indirect oxidation via in situ electroproduced ^•^OH on the anodic surface [15]. Ideal EAOP anodes for ^•^OH generation include doped‐SnO_2_, boron‐doped diamond, and PbO_2_, among which PbO_2_ anodes are particularly promising due to their high oxygen evolution potential (1.9 V vs. SCE) and low cost [16, 17, 18]. However, drawbacks with respect to mass transfer limitations, insufficient current efficiency, and stability issues may lead to unsatisfactory removal performances on PbO_2_ anodes.

Heteroatom modification is an effective strategy for addressing these limitations. Dopants such as Co, Al, Ti, In, and Cu can regulate PbO_2_ crystal growth, coating compactness, electronic conductivity, and interfacial charge–transfer behavior [17, 19, 20]. Among these elements, Cu was reported to be capable of improving the electrodeposition nucleation rate of PbO_2_ crystal, reducing the PbO_2_ particles which allows a denser crystal surface [21, 22, 23]. Cu as a cost‐effective dopant possesses the second highest electrical conductivity among metals and allows it serve as a highly suitable candidate for improving the electrochemical behaviors of PbO_2_ electrodes [24, 25]. However, the relationship between Cu‐induced structural/electrochemical changes and CEX degradation remains insufficiently clarified, especially with respect to the relative roles of ^•^OH‐mediated oxidation and anodic direct oxidation under environmentally relevant chloride concentrations.

Herein, we synthesized a Ti/Sn‐SbO_*x*_/Cu‐doped PbO_2_ (Cu/PbO_2_) anode and and evaluated for CEX degradation. This study was designed to clarify four questions: (i) whether Cu modification improves coating compactness, electroactive surface area, oxygen evolution behavior, and charge–transfer kinetics compared with pristine PbO_2_; (ii) how current density, initial pH, and chloride concentration affect CEX removal; (iii) how ^•^OH‐mediated oxidation and anodic direct oxidation contribute to CEX degradation; and (iv) whether the modified anode maintains its performance in actual pharmaceutical wastewater. The Cu/PbO_2_ system was also benchmarked against representative CEX removal technologies to clarify its advantages and remaining limitations.

## 2 Materials and Methods

### 2.1 Chemicals and Materials

Cefalexin (C_16_H_17_N_3_O_4_S), sodium sulfate (Na_2_SO_4_), sulfuric acid (H_2_SO_4_), sodium hydroxide (NaOH), sodium chloride (NaCl), copper nitrate trihydrate (Cu(NO_3_)_2_ · 3H_2_O), lead nitrate (Pb(NO_3_)_2_), potassium fluoride (KF), tert‐butanol (*t*‐BA), methanol (MeOH), and oxalic acid (OA) were all analytical reagent, and they were all obtained from Aladdin Reagent (China) Co., Ltd. The titanium plate was purchased from Yilida metal mesh Co., Ltd., in the local market. The applied cathode was the ruthenium–iridium electrode. The wastewater samples studied in practical application were picked from a cefalexin manufacturer in China. All solutions we studied in experiments were prepared using the deionized water (DI, 18.2 MΩ cm) in Milli‐Q system HX 7040 system.

### 2.2 Fabrications of PbO_2_ and Cu/PbO_2_ Anodes

The titanium plate (60 × 30 mm) was polished, alkaline washing was done with NaOH, and acid etching was done before being applied for electrodes synthesis. The Sn–SbO_*x*_ intermediate layer was coated and calcinated at 500 °C on the pretreated titanium substrate. This intermediate layer was introduced to improve coating adhesion and interfacial conductivity and suppress Ti substrate passivation. PbO_2_ was then electrodeposited from a Pb(NO_3_)_2_/KF acidic bath with an initial pH of 1.5 – 2.5. For the Cu/PbO_2_ electrode, 0.005 mol L^−1^ Cu(NO_3_)_2_ · 3H_2_O was added to the electroplating bath containing 0.5 mol L^−1^ Pb(NO_3_)_2_ and 0.03 mol L^−1^ KF. For clarity, the final electrodes are denoted as PbO_2_ and Cu/PbO_2_ throughout this manuscript, although both were prepared on the Ti/Sn–SbO_*x*_ substrate/intermediate structure [23, 26].

### 2.3 Morphological Characterization and Electrochemical Measurements

Morphological characteristics of the surfaces at electrodes were revealed via scanning electron microscopy (SEM, Hitachi S4700, Japan). All the electrochemical performances were analyzed via an electrochemical workstation (Autolab PGSTAT302 N, Switzerland) with a three‐electrode system. The working electrodes in the three‐electrode system were PbO_2_ and Cu/PbO_2_, a saturated calomel electrode (SCE), and a platinum electrode as a reference electrode and a counter electrode, respectively. The linear sweep voltammetry (LSV) technique was applied to investigate the oxygen evolution potential of the electrodes, which was conducted in 1 M KOH. The LSV curves were obtained at an interval of 10 mV/s from 0 to 2.0 V. Cyclic voltammetry (CV) measurements were utilized to evaluate the electroactive surface area, and the CV plots were recorded at 100 mV/s. Electrochemical impedance spectroscopy (EIS) was used to examine the resistance of electrodes. Unless otherwise stated, potentials were converted to the reversible hydrogen electrode (RHE) scale according to *E*_RHE_ = *E*_SCE_ + 0.244 + 0.059 pH.

### 2.4 Analytical Methods

The residual cefalexin concentration in solution in electrochemical degradation experiments was determined via a high‐performance liquid chromatography (HPLC, Agilent, America) equipped with an ultraviolet detector. All the samples were separated through Agilent ZORBAX Eclipse Plus C18 column (150 × 4.6 mm), the mobile phase consisted of methanol and water at a volume ratio of 3:7 with a flowing rate of 1.0 mL/min [27]. The selected ultraviolet detector was set at *λ* =  261 nm and the temperature was 30 °C.

### 2.5 Service Lifetime Tests

The stability was investigated in a three‐electrode setup using the same electrochemical workstation in electrochemical characterizations (See Method 2.3). The potential–time curves of PbO_2_ and Cu/PbO_2_ anodes were recorded in 1 M H_2_SO_4_ under 0.5 A cm^−2^.

## 3 Results and Discussion

### 3.1 Morphology Characterization of PbO_2_ and Cu/PbO_2_ Anodes

**FIGURE 1:**
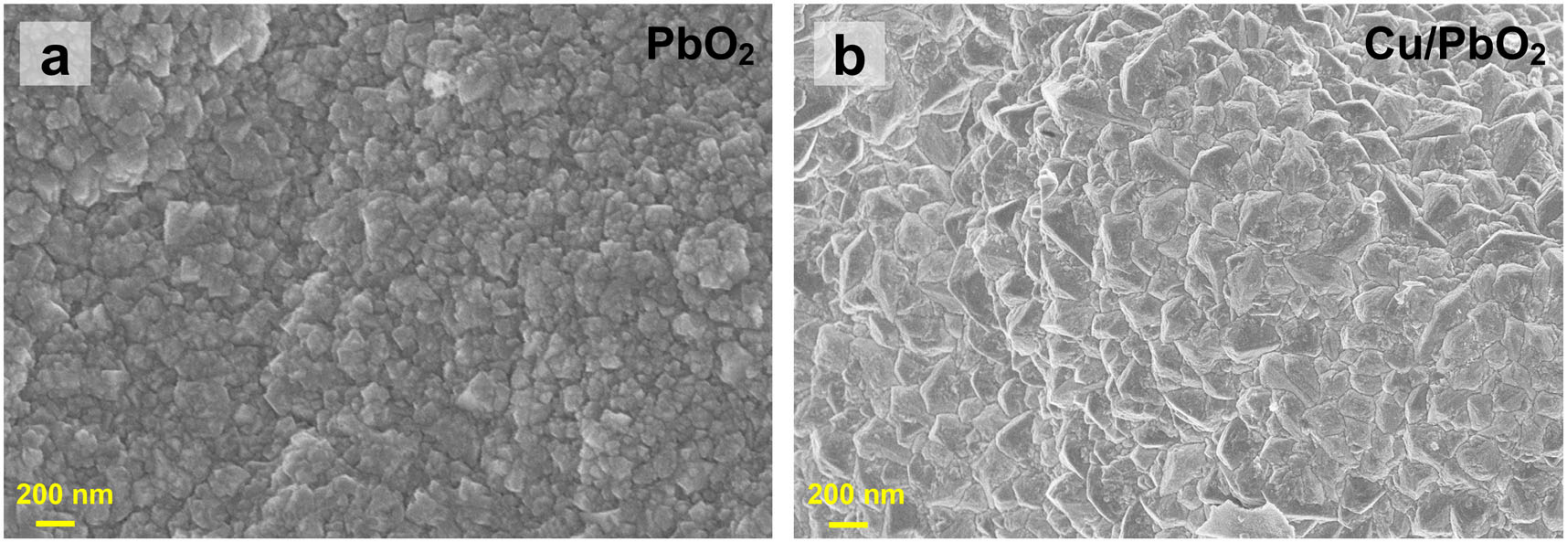
The SEM images of (a) PbO_2_ and (b) Cu/PbO_2_ anodes.

The SEM micrographs of PbO_2_ and Cu/PbO_2_ anodes are given in Figure 1. Compared to the pristine PbO_2_, it could be observed that Cu/PbO_2_ presented the smaller and more compact crystal structure. According to the previous research, a compact surface would largely hinder the TiO_2_ formation from oxidation reaction between Ti substrate and O_2_ [28]. The resistant TiO_2_ was reported that it would exfoliate PbO_2_ from the Ti substrate, reducing the reusability of electrodes [29]. These results implied the corrosion resistance ability via Cu doping into PbO_2_, implying the superior electrochemical persistence of Cu/PbO_2_ anodes.

### 3.2 Electroactivity Analysis of PbO_2_ and Cu/PbO_2_ Anodes

**FIGURE 2:**
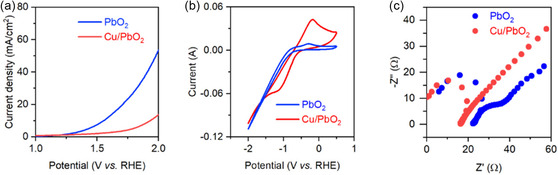
The electrochemical performance curves in 1 M KOH electrolyte. (a) LSV analyses of PbO_2_ and Cu/PbO_2_ anodes. (b) CV curve of PbO_2_ and Cu/PbO_2_ anodes with a scan rate at 100 mV s^−1^. (c) Nyquist plot of PbO_2_ and Cu/PbO_2_ anodes.

Organic pollutants in EAOPs are generally degraded through direct anodic oxidation and indirect oxidation by ROS. Direct oxidation proceeds through electron transfer from the pollutant to the anode surface, whereas indirect oxidation mainly relies on surface‐bound hydroxyl radicals (M(^•^OH)) generated from water oxidation. The ^•^OH was formed through H_2_O oxidation as Equations (1) and (2) illustrated [30, 31]. Because the oxygen evolution reaction (OER) competes with ^•^OH‐mediated pollutant oxidation (Equations (3) and (4)), a higher OER onset potential usually indicates a lower tendency toward parasitic oxygen evolution and a greater opportunity for electrogenerated oxidants to participate in pollutant oxidation [32, 33, 34, 35, 36]. As we could observe, the LSV curves of PbO_2_ and Cu/PbO_2_ anodes showed that the onset potentials of OER were 1.15 and 1.38 V versus RHE, respectively (Figure 2a). The positive shift observed for Cu/PbO_2_ anode suggested that Cu modification suppressed OER and favored the utilization of electrogenerated oxidants for CEX degradation [17].



(1)

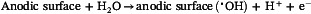






(2)

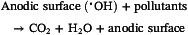






(3)

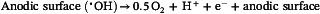






(4)

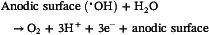




The electroactive surface areas of Cu/PbO_2_ and PbO_2_ anodes were related to the number of active sites on the anodes, which could be reflected by voltammetric charge quantity (q*) in CV graphs [37, 38]. q* was calculated with Equation (5).

(5) q∗ =∫idV/v

where *∫ idV* was an indicator of the integral area of CV curves, and *v* was the selected scan rate at 100 mV s^−1^ [17]. As shown in Figure 2b, the q* of the Cu/PbO_2_ anode was 2.35‐fold higher than that of PbO_2_ anode (calculated through the integral area), indicating that Cu modification increased the electroactive surface area and provided more accessible active sites. Additionally, CV plots displayed in Figure 2b showed a minor oxidation peak at −0.31 V versus RHE in PbO_2_ anode, while a more significant oxidation peak which shifted positively toward higher potential at −0.18 V versus RHE occurred in Cu/PbO_2_ anode (Figure 2c). The stronger oxidation peak current suggested that the cefalexin degraded near the vicinity of Cu/PbO_2_ anode [39]. Furthermore, the curve of Cu/PbO_2_ anode showed an extra additional oxidation peak at around −1.5–−1.0 V versus RHE, indicating that direct oxidation (through electron transfer) on the electrode participated in cefalexin decomposition [17]. Overall, the CV results support the coexistence of direct anodic oxidation and indirect ^•^OH‐mediated oxidation on Cu/PbO_2_ anode, which implied both contributions of direct and indirect (through ^•^OH) for cefalexin decomposition and mineralization.

The charge transfer rates/kinetics between electrode and solution was also evaluated via EIS tests. EIS plots of Cu/PbO_2_ and PbO_2_ anodes are given in Figure 2c, where two semicircles are present in the high‐frequency region. The radius of semicircles in Cu/PbO_2_ anode was smaller than that of PbO_2_ anode, indicating a lower charge transfer resistance at the electrode–solution interface and a higher onset potential of OER in Cu/PbO_2_ anode [34, 40]. The equivalent circuit was used to obtain detailed impendence data via Zview2 software, where *R*_s_ stood for solution resistance between the counter electrode and working electrode. *R*_ct_ represents charge transfer resistance. CPE is a descriptor of capacitance, and *W* is the charge diffusion resistance in solution. The fitting curve of Cu/PbO_2_ anode showed that *R*_ct_ was 17.5 Ω/cm^2^, which was 1.21‐fold smaller than that of PbO_2_ anode (21.2 Ω/cm^2^). This decrease in *R*_ct_ demonstrates that Cu modification improved the conductivity and interfacial electron‐transfer kinetics of PbO_2_. Together with the increased q* and higher OER onset potential, these results explain the enhanced activity of Cu/PbO_2_ in subsequent CEX degradation experiments.

### 3.3 Effect of Current Density on Cefalexin Removal

**FIGURE 3:**
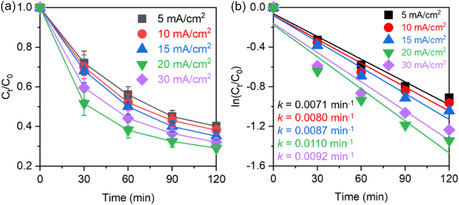
(a) The effect of current density for cefalexin removal via Cu/PbO_2_ anode and its corresponding rate constants (b). Experimental conditions: [cefalexin]_0_ = 1 mM, [Na_2_SO_4_] = 100 mM, and *j* = 5, 10, 15, 20, and 30 mA cm^−2^.

After confirming the improved electrochemical reactivity of Cu/PbO_2_, the effect of current density on CEX removal was examined from 5 to 30 mA cm^−2^ (Figure 3a). The degradation data were fit using the apparent pseudo‐first‐order model ln(*C*_0_/*C*_t_) = *k* *t* (Figure 3b and Table 2). This model is used as an empirical kinetic description commonly applied in EAOPs when the target pollutant concentration is low relative to the oxidant‐generating flux, and short‐lived radicals rapidly reach a quasi‐steady state. Therefore, the *k* values should be interpreted as apparent rate constants rather than elementary kinetic constants. CEX removal increased with current density up to 20 mA cm^−2^, reaching 70.9 ± 1.2%, but decreased when the current density was further increased to 30 mA cm^−2^. The corresponding apparent rate constants were 0.0071, 0.0080, 0.0087, 0.0110, and 0.0092 min^−1^ at 5, 10, 15, 20, and 30 mA cm^−2^, respectively.

**TABLE 2:** Apparent pseudo‐first‐order kinetic parameters for CEX degradation at different current densities using the Cu/PbO_2_ anode.

Current density, mA cm^−2^	Removal, %	*k*, min^−^^1^	*R* ^2^
5	59.8 ± 2.1	0.0071	0.98
10	61.7 ± 1.8	0.0080	0.99
15	64.6 ± 1.4	0.0087	0.98
20	70.9 ± 1.2	0.0110	0.98
30	72.3 ± 0.4	0.0092	0.99

The cefalexin removals and rate constants at 20 and 30 mA cm^−2^ were 1.19 and 1.56‐fold and 1.56 and 1.24‐fold higher than those of 5 mA cm^−2^. Notably, the optimal current density is found to be at 20 mA cm^−2^. These results indicated that larger current density promoted the contaminant degradation and simultaneously enhanced the OER, showing the competition with ^•^OH production and organic compounds degradation at anodic surfaces, which finally reduced the overall contaminants' removal performances of the system [41]. For instance, Zhang and coworkers reported that increasing current density from 2 mA cm^−2^ to 8 mA cm^−2^ could improve imidacloprid‐containing wastewater treatment, but the pollutant decomposition performance declined when current density increased to 10 mA cm^−2^ [33]. Wei and colleagues found that chemical oxygen demand (COD) removal was more effective at 30 mA cm^−2^ compared with that at 50 mA cm^−2^. They deduced the side reactions, possibly H_2_O breakdown for H_2_ and O_2_ evolution, posing a threat to a deleterious effect on the overall current efficiency [42]. In addition to promoting the OER, higher current density also converts ^•^OH into ^•^HO_2_ (Equations (6) and (7)), which had a weaker ability for pollutants degradation [43].



(6)

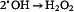






(7)

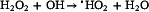




Overall, we confirmed the optimal current density at 20 mA cm^−2^ for cefalexin decomposition.

### 3.4 Effect of Initial pH on Cefalexin Removal

**FIGURE 4:**
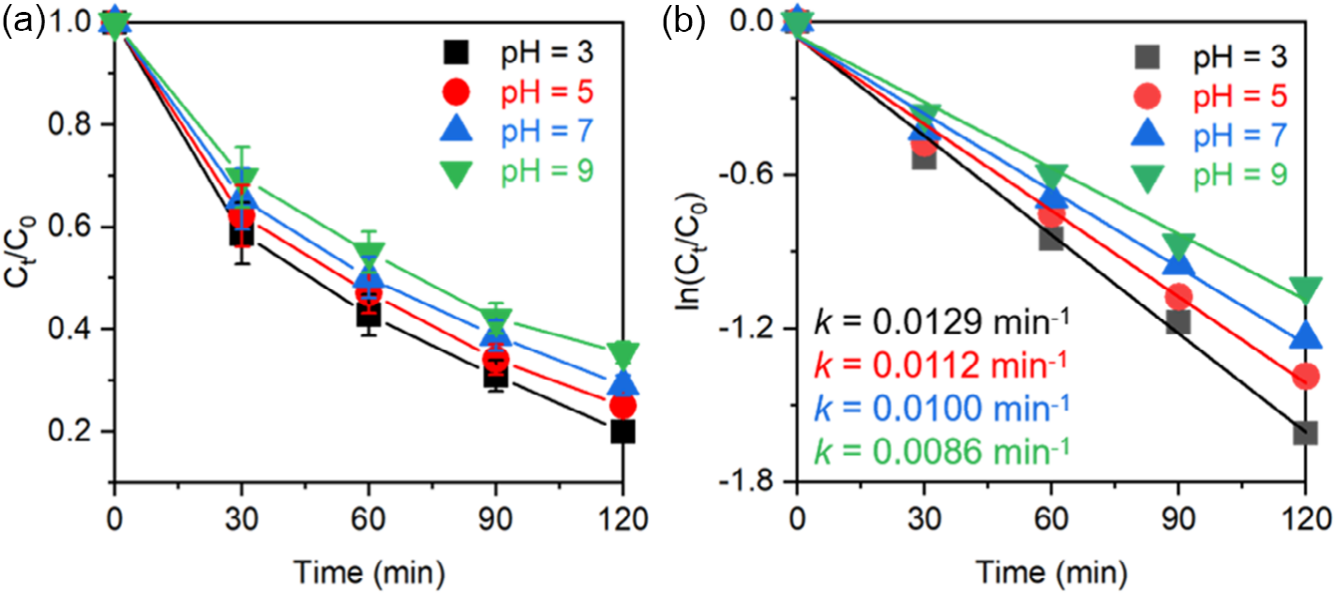
(a) The effect of initial pH for cefalexin removal via Cu/PbO_2_ anode and its corresponding rate constants (b). Experimental conditions: [cefalexin]_0_ = 1 mM, [Na_2_SO_4_] = 100 mM, and *j* = 20 mA cm^−2^, initial pH = 3, 5, 7, 9.

The effect of solution pH for cefalexin degradation in Cu/PbO_2_ anode was also conducted due to its important role in influencing the ionization of pollutants [17, 44]. Hence, the cefalexin removal under various pH ranging from 3 to 9 was investigated. As illustrated in Figure 4a, the removal efficiency of cefalexin reached the highest at 80.2 ± 1.1% at pH = 3, while the efficiencies decreased to 75.2 ± 1.6%, 70.1 ± 1.3%, and 64.5 ± 0.5% at pH = 5, 7, and 9, respectively. The reaction rate constants of cefalexin decomposition in Cu/PbO_2_ anode were 0.0129, 0.0112, 0.0100, and 0.0086 min^−1^ at pH = 3, 5, 7, and 9, respectively (Figure 4b). These results indicate that the contaminant oxidation efficiencies reduce as pH increases, and acidic conditions were more conducive to cefalexin removal [45]. Many researches reported similar phenomena. For instance, Sharan and coresearchers pointed out that lower initial pH was more efficient for ofloxacin degradation during the electrochemical oxidation process [39]. Mandal and colleagues found that 2,4‐dinitrophenol removal efficiency declined with increasing pH [46]. This was mainly attributed to the lower conductivity of solution and lower onset potential OER under higher pH [47, 48], as well as the elimination reaction of OH^−^ (Equation (8)) [49], causing the suppression of ^•^OH production for cefalexin decomposition [48]. Furthermore, it was reported that the flow rate of oxygen at anodic surfaces inevitably increased when the oxygen production rose [32, 50]. The abundant oxygen at anode surfaces would decrease the contaminant diffusion between solution and anodic surface [50, 51].



(8)

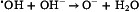




### 3.5 Effect of Different Concentrations of Chloride Ion on Cefalexin Removal

**FIGURE 5:**
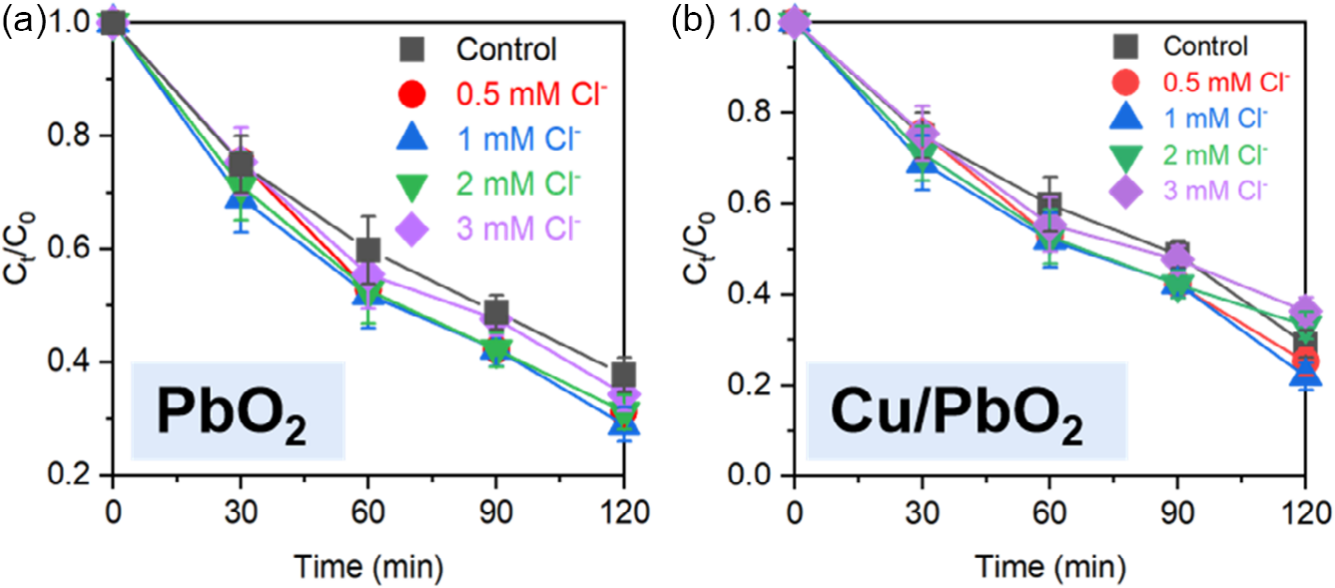
The effect of Cl^−^ for cefalexin removal. (a) PbO_2_ anode. (b) Cu/PbO_2_ anode. Experimental conditions: [cefalexin]_0_ = 1 mM, [Na_2_SO_4_] = 100 mM, *j* = 20 mA cm^−2^, [Cl^−^] = 0.5, 1, 2, and 3 mM.

Chloride ion (Cl^−^) is the most common water matrix in aquatic environment. We investigated the effects of cefalexin degradation under different Cl^−^ concentrations using PbO_2_ and Cu/PbO_2_ anodes (Figure 5a,b). In both PbO_2_ and Cu/PbO_2_‐mediated electrochemical systems, it could be found that 0.5–1 mM Cl^−^ promoted the oxidation of cefalexin, however; 2–3 mM Cl^−^ exhibited the adverse effect for cefalexin degradation. The decomposition of cefalexin at 62.2% ± 1.7% (without Cl^−^) was observed in the PbO_2_‐mediated electrocatalytic system, which reached 72.2% ± 2.1% (with 0.5 mM Cl^−^) and 74.4 ± 2.3% (with 1 mM Cl^−^) and dropped to 66.8% ± 1.0% (with 2 mM Cl^−^) and 63.6 ± 0.7% (with 3 mM Cl^−^). Regarding the Cu/PbO_2_ electrocatalytic system, the cefalexin degradation increased from 70.9 ± 1.2% (without Cl^−^) to 74.8 ± 1.9% (with 0.5 mM Cl^−^) and 78.0 ± 0.7% (with 1 mM Cl^−^), while the removal rates declined to 70.0 ± 3.0% (with 2 mM Cl^−^) and 68.6 ± 1.6% (with 3 mM Cl^−^). These results confirm that different levels of Cl^−^ concentrations had different influences on contaminant removal.

This nonmonotonic trend can be explained by the dual role of chloride. A small amount of Cl^−^ probably reacted with ^•^OH and generated Cl^•^ and Cl_2_^•−^ (Equations (9)–(11)) [52, 53], although Cl^•^ and Cl_2_^•−^ were less reactive species with target pollutants than that of ^•^OH [54]. The extra Cl^•^ and Cl_2_^•−^ could subsequently be utilized to degrade cefalexin. At higher concentrations, however, excess Cl^−^ can compete with CEX and water oxidation for anodic surface sites and rapidly scavenge ^•^OH, shifting the oxidant pool from highly reactive ^•^OH toward chlorine‐centered radicals that may be less effective for CEX oxidation under the present conditions. High Cl^−^ levels may also enhance side reactions such as chlorine evolution and hypochlorite formation and may increase the risk of chlorinated byproducts. Therefore, the decrease in removal efficiency at 2–3 mM Cl^−^ is not contradictory to active‐chlorine chemistry; instead, it reflects a transition from beneficial co‐oxidation at low Cl^−^ concentration to radical scavenging, surface competition, and side reactions at higher Cl^−^ concentrations.



(9)

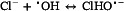

(10) $${\mathrm{ClHO}}^{•-}\;+\;H^{+}\;\to \;{\mathrm{Cl}}^{•}\;+\;H_{2}O$$

(11) $${\mathrm{Cl}}^{•}\;+\;{\mathrm{Cl}}^{‐}\;↔\;{{\mathrm{Cl}}_{2}}^{•-}$$

### 3.6 Degradation Pathway Identification, Stability, and the Practical Application of PbO_2_ and Cu/PbO_2_ Anodes

**FIGURE 6:**
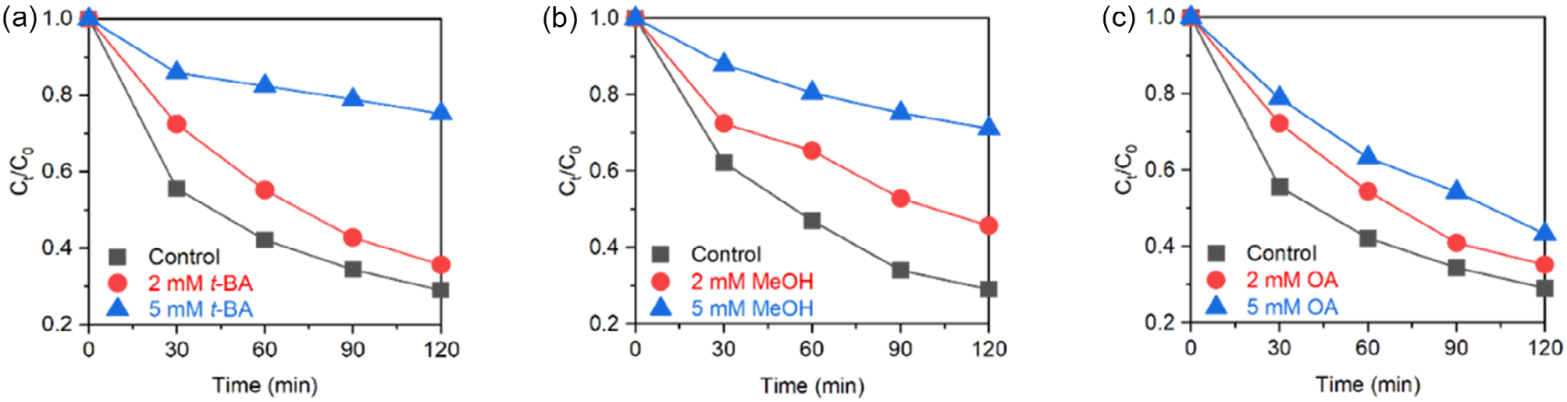
The inhibition of cefalexin removal via different scavengers using Cu/PbO_2_ anode. (a) *t*‐BA. (b) MeOH. (c) OA. Experimental conditions: [cefalexin]_0_ = 1 mM, [Na_2_SO_4_] = 100 mM, *j* = 20 mA cm^−2^.

To identify the dominant oxidation pathway for cefalexin removal, we employed quenching experiments to confirm the ROS. Tertiary butanol (*t*‐BA)and methanol (MeOH) were added as ^•^OH quenching agents during cefalexin electrochemical oxidation in the Cu/PbO_2_‐mediated electrochemical system [39]. As Figure 6a shows, *t*‐BA significantly affected the cefalexin degradation efficiencies, diminishing the removal rate from 70.9% to 64.3% (2 mM *t*‐BA) and to 24.8% (5 mM *t*‐BA). The degradation of cefalexin was also suppressed when MeOH was added, exhibiting the reduction of cefalexin removal from 70.9% to 54.3% (2 mM MeOH) and to 28.8% (5 mM MeOH) (Figure 6b). OAOA was employed as the probe to identify the contribution of direct oxidation for cefalexin degradation. As shown in Figure 6c, cefalexin removal reached 70.4% and 64.7% with 2 and 5 mM OA (0.007 and 0.005 min^−1^), indicating minor contribution of direct oxidation on the anodic surface. The above results indicate the critical roles of ^•^OH and anodic oxidation in cefalexin decomposition.

**FIGURE 7:**
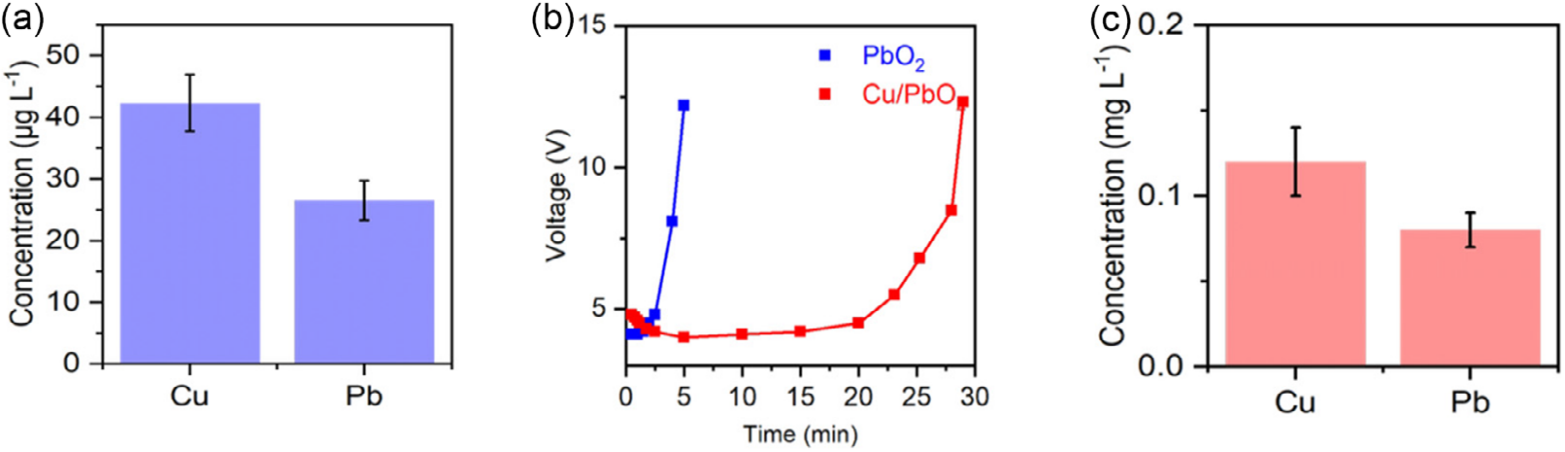
(a) Cu and Pb metal leaching after five consecutive degradation cycles. (b) The accelerated service lifetime tests of PbO_2_ and Cu/PbO_2_ anodes in 1 M H_2_SO_4_ at 0.5 A cm^−2^. The needed time for the cell voltage to reach 10 V was determined as the service lifetime of PbO_2_ and Cu/PbO_2_ anodes. (c) Cu and Pb metal leaching after stability test.

To further evaluate the possible secondary metal release from the Cu/PbO_2_ anode, Cu and Pb concentrations in the electrolyte were measured after electrolysis by inductively coupled plasma–optical emission spectrometry (ICP–OES). After five consecutive degradation cycles, the Cu and Pb concentrations slightly increased to 42.3 ± 4.6 and 26.5 ± 3.2 µg L^−1^, respectively, indicating limited metal leaching during repeated operation (Figure 7a). The stability of PbO_2_ and Cu/PbO_2_ anodes was another essential indicator of electrode properties, which were examined by accelerated service lifetime tests. In accelerated service lifetime tests, the electrodes would be regarded as invalid when the applied voltage was beyond 10 V [55]. It could be found that the stability of Cu/PbO_2_ anode was much higher than that of PbO_2_ anode (Figure 7b), exhibiting the service lifetime at 5.1 h and 12.2 h, respectively (2.39‐fold improvement). The reason for its enhanced stability and efficiency was probably due to the employment of Cu to modify PbO_2_. Specifically, Cu doping was reported to be capable of preventing the electrolyte from reacting with the Ti substrate and relaxing the strains accumulated in the coating layers, which thus exerted a beneficial effect on the stability of the Cu/PbO_2_ anode [56].

Additionally, Cu doping also has advantages on conductivity which has been proven via the lowered impendence result, and the improved conductivity reduces the interference active species generation [56]. In contrast, higher Cu and Pb release was observed after the accelerated service‐life test, reaching 0.12 ± 0.02 and 0.08 ± 0.01 mg L^−1^, respectively (Figure 7c), which can be attributed to the much harsher testing conditions of 1 M H_2_SO_4_ and 0.5 A cm^−2^. These results suggest that the Cu/PbO_2_ anode maintained good structural stability under normal degradation conditions, although long‐term metal release should still be carefully monitored before practical application.

**FIGURE 8:**
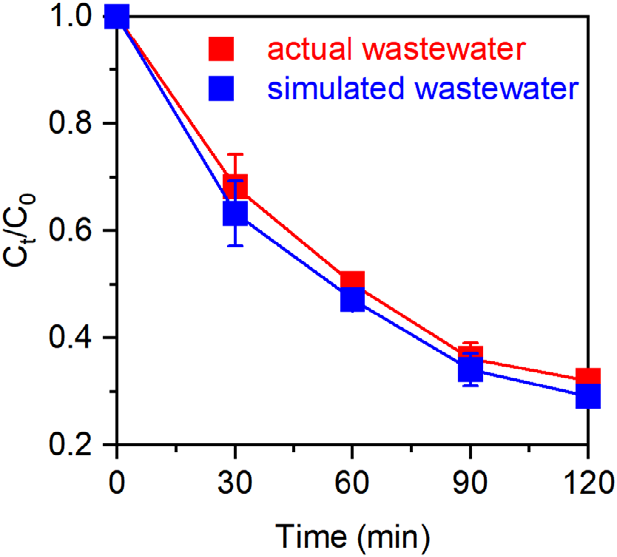
The comparisons of removal performance of Cu/PbO_2_ anode in simulated wastewater and actual wastewater containing cefalexin.

To evaluate practical feasibility, CEX degradation was compared in simulated wastewater and actual wastewater collected from a CEX manufacturer (Figure 8). The Cu/PbO_2_ anode achieved 68.2 ± 2.1% CEX removal in actual wastewater, close to the 70.9 ± 1.2% obtained in simulated wastewater. The slight decrease in the actual matrix was likely caused by competition from coexisting organic matter and inorganic ions for •OH and active sites. To make this comparison more transparent, the key physicochemical properties of the actual wastewater are reported in Table 3, including pH, conductivity, COD, total organic carbon (TOC), Cl^−^, SO_4_^2−^.

**TABLE 3:** Physicochemical characteristics of the actual CEX wastewater used in this study.

Parameter	Value
pH	7.5
Conductivity (mS cm^−1^)	15
COD (mg L^−1^)	100
TOC (mg L^−1^)	250
Cl^−^ (mM)	2.1
SO_4_^2−^ (mM)	10

### 3.7 Strengths and Limitations

Compared with pristine PbO_2_, the Cu/PbO_2_ anode showed clear advantages, including denser coating morphology, larger electroactive surface area, lower charge–transfer resistance, higher OER onset potential, improved CEX removal, and longer accelerated service life. The process also avoids continuous addition of chemical oxidants because reactive species are generated electrochemically. However, several limitations should be acknowledged. (i) Only CEX was selected as the target contaminant, so applicability to mixed‐antibiotic wastewater requires further validation. (ii) Cu and Pb leaching during long‐term operation were not quantified, which is essential for assessing secondary pollution, electrode deactivation, and the possibility that the used electrode gradually loses Cu‐related functionality. (iii) Transformation products, toxicity evolution, chlorinated byproducts in chloride‐containing systems, and mineralization were not systematically analyzed. (iv) The current cost discussion remains qualitative at the laboratory scale; accurate operating cost requires cell‐voltage profiles, treated volume, electrode lifetime under realistic conditions, and local electricity price. These limitations identify the most important next steps for practical scale‐up and environmental‐risk assessment.

## 4 Conclusion

This study demonstrates that Cu modification is an effective strategy for improving the activity and durability of PbO_2_ anodes for electrochemical CEX degradation. Compared with pristine PbO_2_, Cu/PbO_2_ exhibited a denser morphology, a 2.35‐fold higher voltammetric charge, a higher OER onset potential, and a lower *R*_ct_ (17.5 vs. 21.2 Ω cm^2^), all of which contributed to enhanced pollutant oxidation. The optimum current density for the Cu/PbO_2_ system was 20 mA cm^−2^, at which CEX removal reached 70.9 ± 1.2% and the apparent rate constant was 0.0110 min^−1^. Acidic conditions improved degradation, while chloride showed a concentration‐dependent dual effect: 0.5 – 1.0 mM Cl^−^ enhanced removal, whereas 2 – 3 mM Cl^−^ inhibited it because of radical scavenging, surface competition, and side reactions. Quenching experiments confirmed that •OH‐mediated oxidation was the dominant pathway, with a secondary apparent contribution from anodic direct oxidation or other nonquenched pathways. Cu/PbO_2_ also showed a 2.39‐fold longer accelerated service life than PbO_2_ and maintained comparable performance in actual wastewater. These results indicate that Cu/PbO_2_ is a promising anode for antibiotic wastewater treatment, while future work should quantify metal leaching, mineralization, transformation products, toxicity evolution, and full energy/cost metrics under long‐term operation.

## Funding

This work was supported by The Natural Science Foundation of the Jiangsu Higher Education Institutions of China (22KJB610020).

## Conflicts of Interest

The authors declare no conflicts of interest.

## Data Availability

The data that support the findings of this study are available from the corresponding author upon reasonable request.
